# Diabetes-related foot complications and caregiver burden: insights from a cross-sectional study in multi-ethnic Asians

**DOI:** 10.7189/jogh.16.04166

**Published:** 2026-07-17

**Authors:** Tosha Ashish Kalhan, Babitha Sekar, Gladys Tan Ann Gie, Josip Car, Andy Hau Yan Ho, Kavita Venkataraman

**Affiliations:** 1 National Dental Research Institute Singapore, National Dental Centre, Singapore, Republic of Singapore; 2 Saw Swee Hock School of Public Health, National University of Singapore, Singapore, Republic of Singapore; 3 Faculty of Pharmacy, National University of Singapore, Singapore, Republic of Singapore; 4 Lee Kong Chian School of Medicine, Nanyang Technological University, Singapore, Republic of Singapore; 5 King’s College London, School of Life Course and Population Sciences, London, UK; 6 School of Social Sciences, Psychology, Nanyang Technological University, Singapore, Republic of Singapore

## Abstract

**Background:**

Informal caregivers (unpaid family or friends) of individuals with type two diabetes mellitus (T2DM) may experience substantial subjective, time and financial burden, especially in the presence of diabetes-related lower extremity complications (DRLEC). However, evidence on caregiver burden in Asians is limited. Hence, we aimed to investigate the association between DRLEC and informal caregiver burden, quantify the caregiving time and costs attributable to DRLEC by comparing with those without DRLEC, and evaluate how caregivers’ sociodemographic characteristics influence these associations.

**Methods:**

We enrolled individuals from healthcare institutions in Singapore with T2DM and their caregivers. Caregiving burden was evaluated comprehensively, with both subjective burden and caregiving time assessed through interviewer-administered questionnaires. We measured subjective burden using the brief assessment scale for caregivers (BASC), while the monetary value of caregiving time was estimated using the replacement cost method. DRLEC was defined as current or previous ulceration and/or amputation. Linear regression was used to investigate associations between DRLEC and caregiving burden, adjusting for care recipient-related factors (age, sex, race, occupation, and duration of diabetes), while caregiver-related factors (age, sex, monthly income, working status, and marital status) were assessed as effect modifiers.

**Results:**

A total of 143 informal caregiver-care-recipient dyads were enrolled. Mean age of caregivers was 53.4 years (standard deviation (SD) = 14.1), and 64.3% were females. Mean BASC score was 1.98 (SD = 0.55; range = 0–3). Caregivers reported spending an average of 48 hours/week on caregiving duties. The presence of DRLEC among care recipients was associated with significantly greater caregiver burden (approximately 0.30 points), weekly caregiving hours (approximately 14.27 hours), and annual caregiving cost (approximately SGD 12 520), compared with caregivers of individuals without DRLEC. Associations were stronger among caregivers who were female, employed, married, and had a lower monthly income.

**Conclusions:**

Presence of diabetes-related lower extremity complications was associated with a disproportionately high caregiver burden, particularly in married female caregivers from lower-income households.

Diabetes mellitus poses a significant healthcare challenge with a predicted rise in the number of affected individuals from 537 million to 783 million by 2045 [1]. Complications from diabetes have a significant impact on morbidity and mortality and can inflict negative effects on patients’ mobility, mental health, socioeconomic status, and quality of life [2-5], causing patients to require substantial levels of care. As the number of individuals with type two diabetes and its related comorbidities continues to rise, the need for caregivers to provide essential support in managing this chronic condition is likely to become increasingly critical.

Caregiver burden, a key measure of negative caregiving outcomes [6], reflects the mental strain arising from caregiving duties and serves as an independent risk factor for decline in caregivers’ mental health [7] and increased mortality [8]. High caregiver strain has also been linked to poor self-care and medication adherence, especially among caregivers with chronic conditions [9]. This concern is particularly pronounced in those managing individuals with diabetes related lower extremity complications (DRLEC). The need for intensive wound care, assistance with mobility, and management of associated comorbidities contributes to stress, emotional strain and time commitment issues in caregivers [10]. Additionally, posttraumatic psychological stress in caregivers of amputees has been shown to mediate the association between baseline caregiver stress and their subsequent burden and quality of life [11], highlighting the need to formulate early and effective interventions to alleviate the caregiver burden [12].

A substantial portion of caregiving is shouldered by informal caregivers (unpaid family members or friends), who provide ongoing assistance with activities of daily life and diabetes management. Hence, quantifying the time invested in caregiving offers insight into the trade-offs caregivers make, including the frequent neglect of their own healthcare needs. Furthermore, quantifying the economic value of unpaid informal caregiving is crucial for effective long-term care planning, recognising its substantial but often unaccounted-for value, and enabling comprehensive economic evaluations that inform policy and health interventions [13]. However, empirical data on the time and financial demands placed on informal caregivers remain scarce [14,15], limiting a comprehensive understanding of the societal cost of diabetes-related complications and the essential role of caregiving in chronic disease management.

Singapore is a city-state in Southeast Asia, with a multi-ethnic population comprising mainly Chinese, Malay, and Indian individuals. Like many other countries, Singapore is rapidly ageing, with 21% of the population expected to be aged ≥65 years by 2026 [16]. Singaporeans have a one-in-three lifetime risk of diabetes and an estimated one million individuals with diabetes by 2050 [17]. Amputation rates are disproportionately high [18], with the first DRLEC incidence exceeding that reported elsewhere [19]. Furthermore, with informal caregiver burden and associated mental health challenges rising [15], and an estimated annual cost of SGD 1.28 billion (approximately 11% of the government’s healthcare budget) [20], Singapore represents a compelling population for studying the association between caregiver burden and DRLEC [18,19]. However, there has been limited work on the burden on informal caregivers of individuals with DRLEC [21,22], particularly within Asian populations. Hence, we aimed to investigate the association between the presence of DRLEC and caregiver burden; quantify the additional informal caregiving time and costs associated with DRLEC relative to caregivers of patients without DRLEC; and assess the role of caregivers’ socio-demographic characteristics as effect modifiers of these associations in a multi-ethnic Asian population.

## METHODS

### Study design and population

We enrolled individuals diagnosed with type two diabetes mellitus and their informal caregivers. We recruited participants from various hospitals and polyclinics across Singapore, between February and June 2023. We obtained informed consent from all participants. Inclusion criteria for care recipient-caregiver pairs were: care recipient is a Singaporean or permanent resident with type two diabetes mellitus, caregiver is aged ≥21 years, both can read and speak English, Mandarin, or Malay, and both can provide informed consent. All the caregivers were either family members or friends of the care recipients. Formally employed or paid caregivers were excluded from the analysis, as this study focused on informal, unpaid caregiving. This study has been presented according to the STROBE guidelines (Appendix S1 in the **Online Supplementary Document**).

### Data collection

Participants completed a one-time questionnaire as a caregiver or care recipient. Socio-demographic data (sex, age, ethnicity, education, marital and occupational status, income) and the care recipient’s disease-specific data (duration of diabetes, current diabetes treatment, presence of diabetic foot ulcers and amputation history) were self-reported. Primary study exposure was the presence of DRLEC, defined as a current or history of ulceration and/or history of amputation.

### Outcomes

We measured caregiver burden using the 14-item brief assessment scale for caregivers (BASC) questionnaire [23], which captures both adverse and beneficial dimensions of caregiving experience across five domains: negative personal impact (items two, three, four, six, and nine), positive personal impact (items 11, 12, and 14), relationship with other family members (items 10 and 13), medical issues (items seven, eight, and nine), and concern for care recipient (items one and five). Items 1–10 were rated on a four-point frequency scale ranging from ‘not at all’ to ‘a lot,’ while items 11–14 used an agreement scale from ‘agree a lot’ to ‘disagree a lot.’ A ‘does not apply’ response option was available for items 5–14. All items were coded such that lower scores indicated higher caregiver burden. Negatively worded items were reverse-coded to a 0–3 scale (3 = not at all, 2 = a little, 1 = some, 0 = a lot), and positively worded items were coded in the same direction. Overall BASC scores were calculated by averaging available item responses, excluding missing or ‘not applicable’ entries. The resulting mean BASC score ranged from zero to three, with higher scores indicating more positive caregiver outcomes. Primary informal caregivers reported their typical weekly caregiving hours. Caregiving time, measured at a single time point, was annualised for analysis, consistent with previous studies that value informal caregiving time using the wage rate of a close substitute [24]. Using the replacement cost method, the annual caregiving value was estimated by multiplying weekly hours by the 2023 median hourly wage of a full-time healthcare assistant in Singapore (SGD 16.87) and then by 52 weeks, assuming consistent year-round care [25].

### Statistical analyses

We analysed data using Stata, version 17.0 (Stata Corp LLC, College Station, Texas, USA). We reported caregiver burden as the mean and standard deviation (SD), while caregiving time and the corresponding annual monetary estimate were presented as the median and interquartile range (IQR). Multivariable linear regression was used to examine the association between DRLEC in care recipients and caregiver burden, time and estimated annual caregiving cost. Confounding variables in the multivariable model included care recipients’ characteristics such as age, sex, race, occupation, income and duration of diabetes. We used subgroup analysis to explore the role of caregiver-related sociodemographic factors (age, sex, monthly income, working status, marital status) as effect modifiers. We set the level of statistical significance at *P* = 0.05 (two-sided) for all statistical analyses.

## RESULTS

A total of 143 caregiver-care-recipient dyads were enrolled in the study. The mean age of caregivers was 53.4 years (SD = 14.1), and the majority were female (64.3%). Half of the caregivers were Malay (n = 72, 50.3%), followed by Chinese (n = 40, 28.0%) and Indian and others (n = 31, 21.7%). The majority were married (69.9%), and 57.3% reported having secondary education or lower as their highest educational level. They were distributed evenly with regard to employment (53.8% employed, 46.2% not employed), and monthly income levels (53.2%≤SGD 2000 and 46.8%>SGD 2000) (**Table 1**).

**Table 1:** Participants’ characteristics*

Characteristics	Caregivers	Care recipients	*P*-value
Age in years, x̄ (SD)	53.4 (14.1)	68.3 (10.5)	<0.001
Sex			0.02
*Male*	51 (35.7)	72 (50.3)	
*Female*	92 (64.3)	71 (49.7)	
Race			0.57
*Chinese*	40 (28.0)	41 (28.7)	
*Malay*	72 (50.3)	71 (49.6)	
*Indian and others*	31(21.7)	31 (21.7)	
Educational levels			<0.001
*Secondary and below*	82 (57.3)	122 (85.3)	
*Above secondary*	61 (42.7)	21 (14.7)	
Marital status			0.02
*Married*	100 (69.9)	107 (74.8)	
*Not married*	43 (30.1)	36 (25.2)	
Occupational status			<0.001
*Working*	77 (53.8)	20 (14.0)	
*Not working*	66 (46.2)	123 (86.0)	
Monthly income in SGD			0.0008
*<2000*	76 (53.2)	98 (68.5)	
*≥2000*	67 (46.8)	45 (31.5)	

SD – standard deviation, x̄ – mean

*Presented as n (%) unless specified otherwise.

Mean age for care recipients was 68.3 years (SD = 10.5) years with even distribution of male (50.3%) and female (49.7%). Approximately half were Malay (n = 71, 49.6%), followed by Chinese (n = 41, 28.7%) and Indian and others (n = 31, 21.7%). The majority (85.3%) had primary and secondary school as their highest educational attainment, were married (74.8%), unemployed (86.0%), and had a monthly income of<SGD 2000 (68.5%).

The majority of their caregivers were family members or relatives (95.6%). The overall prevalence of DRLEC was 32.2%. Among them, 30 individuals (20.9%) were currently living with both an active or past foot ulcer and a lower-limb amputation, 12 (8.4%) had a history of foot ulceration without amputation, and 4 (2.8%) had undergone lower-limb amputation without a reported history of foot ulceration. Amongst the 34 individuals who underwent limb amputation, most of them had a toe amputation (73.5%), while others had either below-knee (14.7%) or above-knee amputations (11.8%). Overall, care recipients reported living with diabetes for 15.6 (SD = 11.5) years.

Mean BASC score was 1.98 (SD = 0.55), with caregivers reporting an average of 47.6 hours per week on caregiving duties. The average annual cost of caregiving amounted to SGD 41 800.80. On average, caregivers of individuals with DRLEC reported spending twice as much time providing care (60 *vs*. 30 hours/week) and experienced significantly higher caregiving burden (mean score 1.75 *vs*. 2.09) compared to those without DRLEC (**Table 2**). No significant differences were found in mean BASC scores across caregivers' sociodemographic characteristics. However, caregivers with lower education, those who were unemployed, and those with lower incomes reported significantly higher caregiving hours and associated costs.

**Table 2:** Informal caregiver burden, caregiving hours and estimated monetary value by care-recipients’ and caregivers’ socio-demographic characteristics

Characteristics	n	Caregiver burden (BASC score)		Caregiving time (hours/week)	Annual caregiving cost in SGD thousands	*P*-value
		**x̄ (SD)**	***P*-value**	**MD (IQR)**	**MD (IQR)**	
**Care-recipient**						
Presence of DRLEC						
*Yes*	46	1.75 (0.46)	0.0005	60 (30, 82)	52.60 (26.31, 71.93)	0.0004
*No*	97	2.09 (0.56)		30 (10, 70)	26.32 (8.77, 61.40)	
Sex						
*Male*	72	1.95 (0.52)	0.48	50 (20, 80)	43.86 (17.98-70.18)	0.26
*Female*	71	2.01 (0.58)		35 (12, 80)	30.70 (10.52, 70.18)	
Race						
*Chinese*	41	2.01 (0.50)	0.36	24 (10,80)	21.05 (8.77, 70.18)	0.22
*Malay*	71	1.99 (0.54)		50 (21, 80)	43.86 (18.42-70.18)	
*Indian and others*	31	1.92 (0.64)		48 (20, 80)	35.08 (17.54, 70.18)	
Educational levels						
*Secondary and below*	122	1.98 (0.56)	0.81	41 (14, 80)	35.96 (12.28, 70.18)	0.08
*Above secondary*	21	2.01 (0.51)		28 (10, 50)	24.56 (8.77, 43.86)	
Marital status						
*Married*	107	1.98 (0.55)	0.92	42 (20, 80)	36.84 (17.54, 70.18)	0.16
*Not married*	36	1.97 (0.56)		30 (10, 79)	26.31 (8.77, 69.30)	
Occupational status						
*Working*	20	2.30 (0.49)	0.005	40 (12, 55)	35.08 (10.52, 48.24)	0.48
*Not working*	123	1.93 (0.54)		40 (14, 80)	35.08 (12.28, 70.18)	
Monthly income in SGD						
*<2000*	98	1.90 (0.55)	0.01	50 (18, 80)	43.86 (15.79, 70.18)	0.04
*≥2000*	45	2.15 (0.51)		30 (10, 60)	26.31 (8.77, 52.63)	
**Caregiver**						
Sex						
*Male*	51	1.94 (0.51)	0.48	48 (14, 90)	42.10 (12.28, 78.95)	0.22
*Female*	92	2.00 (0.57)		40 (13, 80)	35.08 (11.40, 70.18)	
Race						
*Chinese*	40	2.04 (0.52)	0.70	22.5 (10, 79)	19.73 (8.77, 69.30)	0.15
*Malay*	72	1.96 (0.54)		49 (22.5, 80)	42.98 (19.74, 70.18)	
*Indian and others*	31	1.96 (0.60)		50 (20-80)	43.86 (17.54, 70.18)	
Educational levels						
*Secondary and below*	82	1.96 (0.54)	0.60	50 (28, 80)	43.86 (24.56, 70.18)	0.003
*Above secondary*	61	2.01 (0.56)		24 (10, 70)	21.05 (8.77, 61.40)	
Marital status						
*Married*	100	1.95 (0.55)	0.41	50 (14, 80)	43.86 (12.28, 70.18)	0.13
*Not married*	43	2.04 (0.54)		30 (12, 60)	26.31 (10.52, 52.63)	
Occupational status						
*Working*	77	1.94 (0.55)	0.37	30 (10, 50)	26.31 (8.77, 43.86)	0.0001
*Not working*	66	2.02 (0.54)		65 (24, 90)	57.02 (21.05, 78.95)	
Monthly income in SGD						
*<2000*	76	1.94 (0.57)	0.40	58 (30, 82)	50.87 (26.31, 71.93)	<0.001
*≥2000*	67	2.02 (0.52)		20 (8, 60)	17.54 (7.01, 52.63)	

BASC – brief assessment scale for caregivers, DRLEC – diabetes-related lower extremity complications, IQR – interquartile range, MD – median

*Estimates represent the monetised value of informal caregiving time, calculated using the 2023 median hourly gross wage of healthcare assistants (SGD 16.87/h).

**Figure 1:**
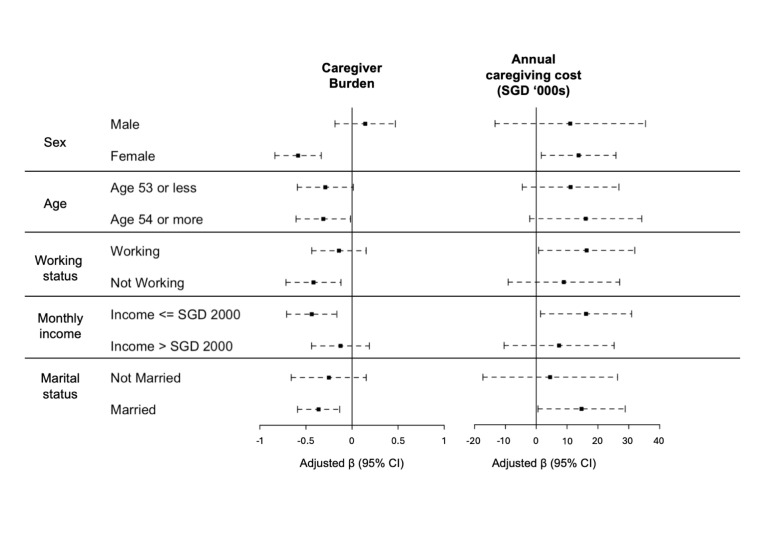
Role of caregiver factors as effect modifiers on the association between DRLEC and caregiver burden and annual caregiving cost. Adjusted for patient-related factors such as sex, age, race, occupational status, income and marital status (n = 143). CI – confidence interval.

Presence of DRLEC was associated with a high caregiver burden (β = –0.30; 95% confidence interval (CI) = –0.50, –0.10, *P* = 0.003), increased caregiving hours (β = 14.27; 95% CI = 1.00, 27.58, *P* = 0.03) and associated caregiving cost (β = 12.52; 95% CI = 0.84, 24.19, *P* = 0.03), compared to those without DRLEC. Subgroup analyses showed that caregiver burden was more pronounced in caregivers who were female, married, non-working or those with a lower monthly income. These findings broadly aligned with higher informal caregiving costs observed in the same subgroups, except for working status, where non-working caregivers reported greater burden but not correspondingly higher costs (**Figure 1**, **Table 3**).

**Table 3:** Association between DRLEC and caregiver burden, caregiving time, and annual caregiving cost

Main model	Caregiver burden (BASC score) (n = 143)		Caregiving time (hours/week) (n = 143)		Annual caregiving cost SGD thousands (n = 143)	
	**Adjusted *β* (95% CI)**	***P*-value**	**Adjusted *β* (95% CI)**	***P*-value**	**Adjusted *β* (95% CI)**	***P*-value**
Presence DRLEC						
*Yes*	–0.30 (–0.50, –0.10)	0.008	14.27 (1.00, 27.58)	0.036	12.52 (0.84, 24.19)	0.036
*No*	ref		ref		ref	

BASC – brief assessment scale for caregivers, CI – confidence interval, DRLEC – diabetes-related lower extremity complications, ref – reference

## DISCUSSION

We comprehensively assessed the subjective burden of care in caregivers of individuals with DRLEC and quantifies the caregiving time and its associated economic costs in a muti-ethnic Southeast Asian population. Our study has two main findings: caregivers of individuals with DRLEC experienced significantly greater burden (approximately 0.31 points lower), with markedly higher caregiving hours (approximately 14.27 hours) and elevated annual caregiving costs (approximately SGD 12 520) and social determinants such as sex, monthly income and marital status of the caregiver significantly influenced the association, with married female caregivers from low-income households reporting greater caregiver burden.

Numerous factors may contribute to higher caregiver burden in individuals with DRLEC, such as significant emotional and financial strain from prolonged healing time, intensive treatments and frequent hospital visits [26], and challenges in providing assistance for daily activities [27] like monitoring glucose levels, administering medications, *etc*. The amount of time spent caregiving is a strong predictor of caregiver stress [28], particularly when the caregiver is a close family member who may feel obligated to provide care [29]. This intense caregiving load with extended caregiving hours can place immense psychological pressure on caregivers, often leading to physical and emotional exhaustion [30]. On the other hand, improved mental health among caregivers of patients with healed ulcers, compared with those of patients with persistent ulcers [31], suggests a direct association between patients’ DRLEC severity and caregivers’ quality of life.

While care recipients were evenly split between the sexes, about two-thirds of caregivers were women, reflecting persistent patriarchal caregiving norms. Gender differences in caregiving for individuals with diabetes have been reported [9,32]. Societal expectations may exacerbate stress, particularly for married working women [33], and this burden is often worsened by low income and limited support [34,35], emphasising the need for gender-specific interventions, such as home environment modifications, to alleviate caregiver stress [36]. Our findings highlight intersectional vulnerabilities among Malay women, whose care recipients had higher rates of foot complications (40.0% *vs*. 27.5% Chinese and 19.4% Indian). These caregivers were predominantly female (72.0%), married (72.0%), less educated (69.0%), and low-income (55.0%), indicating the combined impact of gender, socioeconomic, and cultural factors. Moreover, Malays and Indians spent more weekly hours (50 and 48 hours, respectively) in caregiving compared to Chinese caregivers (24 hours), yet subjective burden scores were similar (mean BASC score was 1.96 *vs*. 2.04), suggesting possible cultural normalisation of caregiving strain where familial roles are socially embedded. These results highlight the need for culturally appropriate caregiver support systems and policy interventions.

Employed caregivers report higher burden, highlighting increased challenges and negative outcomes faced while balancing caregiving and work duties [37]. Although non-working caregivers reported nearly double the weekly caregiving hours (65 *vs*. 30) and annual caregiving costs (SGD 57 020 *vs*. SGD 26 310), subgroup analyses indicated that employed caregivers experienced significantly greater burden (**Figure 1**, **Table 2**). This counterintuitive finding may reflect work – family conflicts that intensify psychological stress and elevate caregiver burden for those balancing employment and caregiving [7].

Our findings highlight a higher burden among low-income caregivers, emphasising the need for more integrated and targeted community-level initiatives that enhance financial security and social support to alleviate this burden [38,39]. In Singapore, initiatives such as the Enhanced Home Caregiving Grant [40], the Caregivers Training Grant [41], NTUC Care Fund [42], Flexible Work Arrangements [40], and Grandparent Caregiver Relief [43] provide financial aid, training, and workplace flexibility to support caregivers. However, significant gaps remain, particularly for female caregivers balancing employment and domestic duties. Targeted community-level initiatives could address gender disparities by expanding paid caregiving leave, improving access to affordable respite care, and providing tailored support for married working women. Additional complementary interventions, such as digital health literacy programs and caregiver-specific financial assistance, should be coupled with robust outcome evaluations to ensure effectiveness and sustainability. Given similar family-centred caregiving norms across many Asian societies, these policy considerations may also be relevant beyond Singapore, particularly in settings seeking to better integrate caregiver support within existing health and social care systems.

The strengths of the study include the use of the BASC index to measure caregiver burden, which is easy to administer and captures adverse and beneficial dimensions of caregiving experience [23], compared to other indices such as the Zarit Burden Interview scale [22,44] and Caregiver Strain Index [45] which predominantly focus on the negative perceptions of caregiving. Second, the dyadic study design provides valuable insights into the caregiver-care recipient relationship in everyday settings, allowing caregiver burden to be contextualised and real-world caregiving dynamics to be captured. Other strengths include accounting for social determinants of both care recipients and caregivers, key predictors of health outcomes [46], providing insights into their influence on caregiver burden in DRLEC.

The cross-sectional design limited our ability to establish causality between caregiver characteristics and burden, warranting longitudinal studies to understand how these factors evolve over time and their impact on caregiver well-being. Although data were collected concurrently from both caregivers and care recipients using validated instruments, allowing partial cross-validation within caregiver-care recipient pairs, some degree of response bias cannot be entirely ruled out. Lastly, variability in care needs between care recipients (within the DRLEC and non-DRLEC groups) over time may influence the time and financial resources required, which could not be assessed in this study owing to the cross-sectional design and should be considered in future studies when comparing groups and extrapolating annual caregiving hours. Additional qualitative studies can provide deeper insights into the specific challenges faced by different caregiver subgroups, informing more tailored support strategies.

## CONCLUSIONS

In conclusion, findings from a multi-ethnic Asian population highlight socio-demographic determinants of the caregivers to influence the association between presence of diabetes-related lower extremity complications and caregiver burden. By identifying vulnerable subgroups of caregivers and understanding the risk determinants that exacerbate their individual burden, healthcare providers can better tailor comprehensive support systems and interventions to enhance caregiver well-being and ultimately improve patient outcomes.

**Data availability:** The data sets generated and/or analysed in this study are available from the authors upon reasonable request.

## Additional material


Online Supplementary Document
